# Sevoflurane decreases self-renewal capacity and causes c-Jun N-terminal kinase–mediated damage of rat fetal neural stem cells

**DOI:** 10.1038/srep46304

**Published:** 2017-04-10

**Authors:** Zeyong Yang, Jingjing Lv, Xingxing Li, Qiong Meng, Qiling Yang, Wei Ma, Yuanhai Li, Zun Ji Ke

**Affiliations:** 1 Department of Anesthesiology, International Peace Maternity and Child Health Hospital, Shanghai Jiao Tong University School of Medicine, 200030, Shanghai, China; 2 Department of Anesthesiology, The First Affiliated Hospital of Wannan Medical College, Wuhu, 241000, Anhui, China; 3 Department of Anesthesiology, First Affiliated Hospital of AnHui Medical University, Hefei, 230022, Anhui, China; 4 Department of Biochemistry, Shanghai University of Traditional Chinese Medicine, Shanghai 201203, China

## Abstract

Increasing studies have demonstrated that sevoflurane can induce neurotoxicity in the developing brains. JNK normally promotes apoptosis. It was hypothesized that sevoflurane affected the proliferation and differentiation of FNSCs and induced cell apoptosis, which caused the learning and memory deficits via JNK pathway. Sevoflurane at a concentration of 1.2% did not induce damage on the FNSC_S_. However, concentrations of 2.4% and 4.8% decreased the cell viability, as shown by the 3-(4,5-dimethylthiazol-2-yl)-2,5-diphenyltetrazolium bromide (MTT) assay, and increased apoptosis, as shown by flow cytometry. The 5-ethynyl-2′-deoxyuridine (EdU) incorporation assay demonstrated that 4.8% sevoflurane reduced the proliferation of FNSCs. Compared with the control group, the 4.8% sevoflurane group showed a decrease in the proportion of undifferentiated FNSCs at 6-h exposure; 4.8% sevoflurane could increase the p-JNK/JNK ratio. JNK inhibition by the specific inhibitor SP600125 enhanced partially the cell viability. Cumulatively, 4.8% sevoflurane induced significant damage on FNSCs; it decreased cell proliferation and proportion of undifferentiated cells as well. JNK pathway might play a key role in the decrease in survival of FNSCs induced by an inhaled anesthetic. The present findings might raise the possibility that JNK inhibition has therapeutic potential in protecting FNSCs from the adverse effects of the inhaled anesthetic.

By definition, fetal neural stem cells (FNSCs) are pluripotent cells with self-renewal capacity that ultimately differentiate into neurons, astrocytes, and oligodendrocytes. Cell death and proliferation are the two determinants of self-renewal capacity. Increasing data suggest that exposure to anesthetics during certain periods of development has long-term deleterious effects on the development of nerves. Anesthetic agents induce cell death, cause synaptic remodeling, and alter the morphology of the developing brain^1^,^2^,^3^,^4^,^5^. Moreover, in humans, children exposed to anesthesia in early life have a higher incidence of learning deficits in adolescence^6^. It is possible that anesthetic effects on FNSCs may mediate some of these morphologic and behavioral phenotypes. Proliferation, differentiation, and migration of cells derived from embryonic FNSCs are critical processes for normal brain development^7^. Most studies on the effects of inhaled anesthetics on hippocampal neurogenesis have focused on isoflurane. Administration of a 50% effective dose concentration of isoflurane to postnatal day 7 rats for 4 h inhibited hippocampal neurogenesis^8^. Anesthetic drugs cause brain cell death and long-term neurocognitive dysfunction in neonatal rats; 6-h isoflurane exposure at or above 1 minimal alveolar concentration (MAC) *in vitro* decreased neural stem cell (NSC) proliferation^9^,^10^. However, a few studies have investigated the effect of the inhalational anesthetic sevoflurane on the apoptosis of FNSCs, which attributed to the depletion of FNSCs and reduction in neurogenesis caused by drugs. Drugs may damage neural cells and cause neuronal deficits, such as cognitive dysfunction and memory impairment. However, the key biological role of JNK in sevoflurane-induced developmental nerve apoptosis remains unknown yet. Also, the precise mechanism underlying the toxic effects on death, proliferation, and differentiation of FNSCs remains largely unknown.

The present study was designed to test the hypothesis that a high dose of sevoflurane might impair proliferation and promote the death of FNSCs, and activate the JNK pathway.

## Materials and Methods

### Cell cultures

Rat FNSCs (Invitrogen, NY, USA, catalog no. R7744–200) were isolated from the cortices of the fetal Sprague–Dawley rats on day 14 of gestation (E14) and cultured according to the manufacturer’s instructions. Briefly, the cells were plated at a density of 50,000 cells/cm^2^ and maintained undifferentiated in StemPro^®^ NSC serum-free medium, supplemented with 48.5 mL of KnockOut Dulbecco’s modified Eagle’s medium/Nutrient Mixture F-12 (Invitrogen, 12660–012), 0.5 mL of 2 mM GlutaMAX-I Supplement (Invitrogen, 35050–061), 20 ng/mL basic fibroblast growth factor (Invitrogen, AA 10–155), 20 ng/mL epidermal growth factor (Invitrogen, PHG0314), and 1 mL of 2% StemPro NSC Neural Supplement (Invitrogen, A10508–01). The FNSCs were plated as an adherent culture by precoating the culture vessels with CELLStart working solution at 37 °C in a humidified atmosphere of 5% CO_2_ in air for 1 h. The culture medium was changed every 48 h, and the cells were detached using prewarmed StemPro Accutase (Invitrogen, A11105–01) and then subcultured when 75–90% confluent. All experiments were carried out on cells between passages 2 and 4 to minimize the experimental deviations. As FNSCs can automatically differentiate into neurons, oligodendrocytes, and astrocytes, the undifferentiated FNSC markers, nestin antibody and sex-determining region Y-box 2 (SOX2) antibody, were used for an immunocytochemical analysis at passage 4 to confirm the proportion of FNSCs used for subsequent assays.

### Anesthetic exposure

FNSCs were exposed to sevoflurane in a gas-tight chamber placed in the incubator at 37 °C, and the concentration of sevoflurane was precisely manipulated via a sevoflurane-specific vaporizer (Yu Yan Instruments, Shanghai, China). The gas mixture contained 5% CO_2_, 21% O_2_, and balanced nitrogen. The treatment groups were put into a large gas-tight chamber of 35 × 25 × 15 cm^3^. The gas mixture contained a certain concentration of sevoflurane, which flowed to the chamber at a rate of 5 L/min for 5 min and then 1 L/min for the remaining exposure time. The concentrations of sevoflurane, O_2_, and CO_2_ in the chamber were checked by infrared absorbance of effluent gas and monitored constantly. The concentrations were maintained throughout experiments using an infrared Ohmeda 5330 agent monitor (Coast to Coast Medical, MA, USA), as in a previous study^11^. The gas-tight chamber itself did not have any effect on cell viability at such a flow rate. The control plates or dishes were placed out of the gas-tight chamber but in the same incubator. After exposure, the plates or dishes were taken out to proceed with the corresponding assays.

### Immunocytochemistry

The cells were fixed with 4% paraformaldehyde in phosphate-buffered saline (PBS) for 15 min, washed three times with 100 μL of 3% bovine serum albumin (BSA) in PBS, and blocked with 100 μL/well 3% BSA in PBS for 20 min at room temperature, followed by incubation with the primary antibodies nestin (Millipore; 1:500 dilution) and SOX2 (Invitrogen, 1:200 dilution) overnight at 4 °C. The wells were then washed three times. Then, the secondary antibody (Alexa FluorR 488 or Alexa FluorR 555, Invitrogen) was applied and incubated for 2 h at room temperature. The wells were washed three times and incubated with 100 μL of 5 μg/mL Hoechst 33342 for 30 min or 5 μg/mL (final concentration) 4,6-diamidino-2-phenylindole (DAPI, Sigma) in PBS for 10 min at room temperature. They were again washed three times with PBS and stored in the dark at 4 °C until image acquisition and statistical analysis.

### Cell viability assay and detection of apoptosis

Cell viability was tested by the 3-(4,5-dimethylthiazol-2-yl)-2,5-diphenyltetrazolium bromide (MTT) assay, as in a previous study^11^. Briefly, FNSCs were seeded at a density of 16,000 cells/well on 96-well plates, and subsequently, 10 μL of MTT per well was added for 4 h in the cell culture incubator immediately after the sevoflurane exposure. The mitochondrial succinate dehydrogenase in viable cells allowed the exogenous MTT to form the water-insoluble formazan and deposit in the cells; this did not occur in the dead cells. The medium was then aspirated after 4 h, and 100 μL/well dimethyl sulfoxide was added to dissolve the purple formazan precipitate, which was quantified spectrophotometrically at 490 nm. Apoptotic cell death was determined using a dead cell apoptosis kit (Invitrogen, V13242) with fluorescein isothiocyanate (FITC) Annexin V and propidium iodide (PI) for flow cytometry according to the manufacturer’s instructions. The cells were harvested after the treatment and washed with cold PBS twice. They were resuspended in 1× Annexin V binding buffer. Then, 5 μL of FITC Annexin V and 1 μL of 100 μg/mL PI working solution were added to each 100 μL of cell suspension and incubated at room temperature for 15 min. After the incubation period, another 400 μL of 1× Annexin binding buffer was added, and the samples were kept on ice until analyzed by flow cytometry.

### Differentiation of FNSCs

FNSCs, growing as an adherent culture in the proliferation medium, were collected 24 h after sevoflurane exposure. They were stained with the primary antibodies SOX2 (Invitrogen, 1:200 dilution) overnight at 4 °C, as described earlier, for an immunohistochemical analysis and then photographed. The total number of SOX2-positive cells was determined.

### 5-Ethynyl-2′-deoxyuridine Incorporation assay and cell proliferation

5-Ethynyl-2′-deoxyuridine (EdU) (Click-iT EdU Alexa Fluor 488 Imaging Kit, Invitrogen) is a nucleoside analog of thymidine. It is incorporated into DNA during active DNA synthesis. Cell proliferation was determined by the EdU incorporation assay, as previously described^10^. Briefly, 100 μL of 2× working solution of EdU was added to the wells with FNSCs in the 96-well plate during the exposure time for 6 hours with different sevoflurane concentrations. After incubation, the cells were fixed with 4% paraformaldehyde for 15 min and then 0.2% Triton X-100 for 20 min at room temperature. The cells in each well were washed twice with 3% BSA between every two steps. Then, 100 μL of Click-iT reaction cocktail was added to each well for 30 min and protected from light. The cells were blocked with 3% BSA for 1 h and then incubated with the anti-nestin antibody (Millipore; 1:500 dilution) overnight at 4 °C. After washing with the 3% BSA, the cells were incubated with the secondary antibody Alexa Fluor 555 for 1 h and then with the 1× Hoechst 33342 solution for 10 min in the dark at room temperature. Each well was washed twice with PBS, and the images were obtained using an Olympus IX-70 inverted fluorescence microscope(Olympus America Inc, Center Valley, PA).

### Image acquisition and analysis

Eight images (number based on previous experience with this system) were acquired per well using an IN Cell Analyzer 1000 (GE Healthcare Life Sciences, CA, USA) in an automated unbiased fashion. This compact bench-top instrument includes an automated Nikon microscope, a high-resolution charge-coupled device camera, a xenon lamp–based illumination, a filter wheel–based wavelength control, and laser-based autofocus and associated image acquisition and analysis software (GE Healthcare). Because image acquisition is automated and large numbers of images can be acquired, cell selection bias is eliminated, and the impact of experimental and biologic variation is reduced^12^. The cells were first identified using top-hat segmentation based on having a nuclear area more than 25 μm^2^. This threshold setting excluded noncellular debris from the analysis. The cells were then outlined based on nestin staining overlapping a nucleus, as defined by staining with Hoechst 33342, using multiscale top-hat segmentation with the characteristic area set at 100 μm^2^ and a sensitivity of 50. For identification and analysis of EdU-positive cells, a two-step filtering process was used. In the first step, nestin-negative cells were excluded; in the second, the threshold setting was used to determine the number of EdU-positive cells. Thus, only nestin-positive cells were analyzed and included in the final analysis. Imaging parameters were set based on a control plate stained with the fluorophore or antibody of interest and the parameters used to image an entire set of matched control and sevoflurane-treated plates. Once the parameters were set, the images were acquired automatically, implying no cell selection bias. In these experiments, eight images were acquired per well using a 20× objective from 8 to 12 wells per treatment condition per assay per time (*N* = 8–12) in matched control and sevoflurane-treated plates. The nuclear intensity threshold for an EdU-positive cell was defined by the intensity of EdU-positive staining in matched control cells. A similar two-step filtering process was used to determine the percentage of cells staining positive for SOX2.

### Statistical analysis

Except PI staining, which failed normality testing, the remainder of the data from each assay at different time points from matched control and sevoflurane-treated cells were analyzed using Prism 5 (GraphPad Software, CA, USA). Two-way analysis of variance (ANOVA) was used with treatment condition and time as the between-group factors and Bonferroni correction for multiple comparisons. Data for one-group variables were analyzed using one-way ANOVA followed by the Tukey’s multiple comparison test. Cell numbers and SOX2 intensity 24 h after treatment were analyzed using an unpaired two-tailed Student *t* test with a Bonferroni correction for multiple comparisons. Data were expressed as mean ± standard deviation. The significance level for all statistical comparisons was set at *P* < 0.05.

## Results

### Confirmation of FNSCs

**Figure 1:**
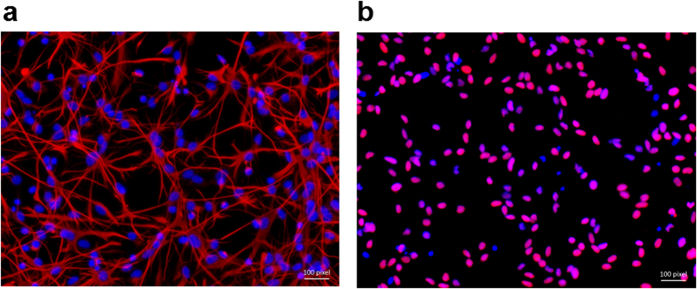
Cells of passage 4 were cultured in the complete NSC proliferation medium, immunostained with nestin (**a**, *pink*) or SOX2 (**b**, *pink*), and then counterstained with Hoechst 33342 (*blue*) as the nuclear marker. More than 90% of the plated cells stained positive for both nestin and SOX2 at passage 4, demonstrating that they were NSCs.

More than 90% of the control cells plated on CELLStart-coated plates stained positive for both nestin and SOX2 at passage 4, confirming that the cells studied in this study were FNSCs (Fig. 1). Cells that were not nestin positive were excluded from the subsequent immunocytochemical analysis.

### Sevoflurane induced FNSCs cytotoxicity in a dose- and time-dependent manner

**Figure 2:**
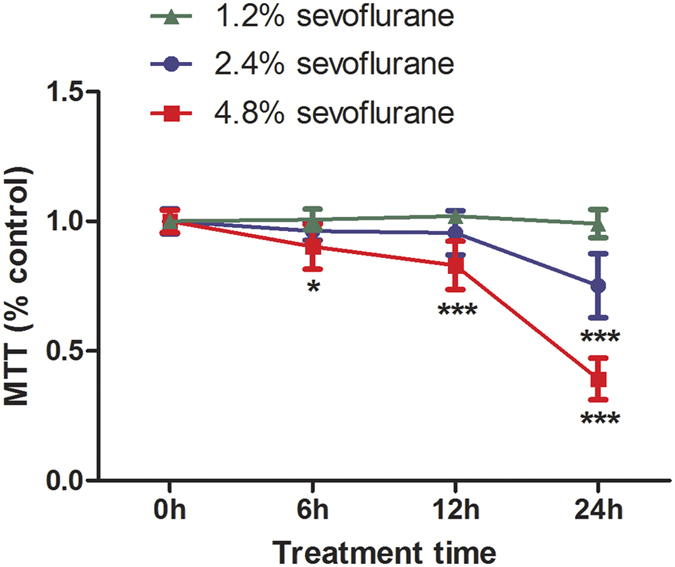
Sevoflurane decreased the viability of rat FNSCs in a dose- and time-dependent manner. Data were obtained from at least four separate cultures, presented as mean ± standard deviation, and analyzed by two-way ANOVA followed by Bonferroni’s multiple comparison tests (*n* ≥ 6 for each condition). *P < 0.05 or ***P < 0.001, respectively, compared with controls. Exposure of FNSCs to 1.2% sevoflurane for 6 h, 12 h, and 24 h had no effect on survival, but exposure to 2.4% sevoflurane, a clinically relevant concentration, resulted in significant cell damage at 24 h (***P < 0.001), as measured by the MTT assay. Exposure to 4.8% sevoflurane for 6 h could decrease cell viability and more significantly for 12 h and 24 h (***P < 0.001).

The activity of FNSCs exposed to sevoflurane was determined by measuring MTT, an initial indicator of cell death, in a quantitative colorimetric assay. The dose- and time-dependence of sevoflurane exposure on the survival of NSCs was determined. The results showed that sevoflurane decreased cell viability in a dose- and time-dependent manner (Fig. 2). The MTT colorimetric assay was used to determine FNSC activity at different concentrations (1.2, 2.4, and 4.8%, respectively) of sevoflurane for 24 h or with different durations (6, 12, and 24 h, respectively) at 4.8% sevoflurane (Fig. 2).

### Sevoflurane induced apoptosis of rat FNSCs

**Figure 3:**
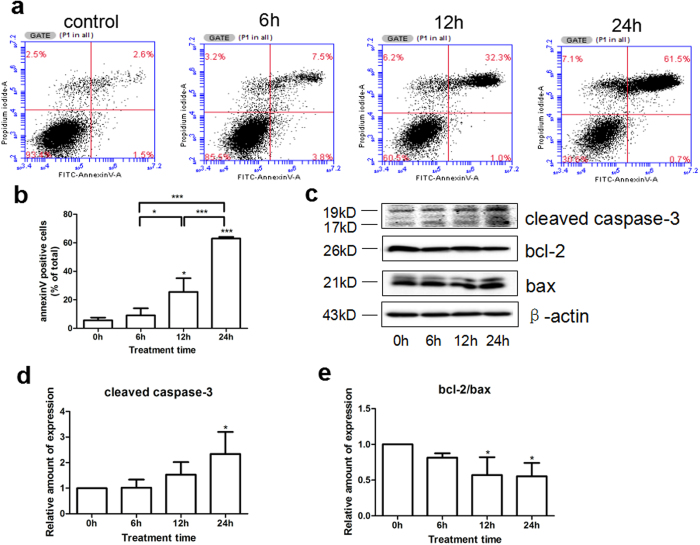
(**a**) FITC Annexin V/PI flow cytometry was used to detect the effects of different durations (6 h, 12 h, and 24 h) of 4.8% sevoflurane on FNSCs-mediated apoptosis. (**b**) Statistical results for Annexin V–positive cells (*P < 0.05, ***P < 0.001). (**c**) Expression levels of apoptosis-executing protein cleaved caspase-3 and apoptosis-related proteins bcl-2 and bax after exposure to 4.8% sevoflurane for different durations. (**d**) Statistical results of the expression of cleaved caspase-3 protein from at least three independent experiments (*P < 0.05). (**e**) Statistical results of the expression of apoptosis-related proteins bcl-2 and bax (*P < 0.05). All data were derived from the results of three independent experiments. *P < 0.05, **P < 0.01, ***P < 0.001.

Annexin V/PI flow cytometry was used to detect the effects of different durations of exposure of 4.8% sevoflurane on apoptosis of rat FNSCs. Western blotting was used to measure the levels of apoptosis-executing protein cleaved caspase-3 and apoptosis-related proteins bcl-2 and bax to further clarify the mechanism of sevoflurane-induced apoptosis. Compared with the control group, treatment with 4.8% sevoflurane for 12 and 24 h led to a time-dependent increase in the number of apoptotic cells (*P* < 0.05 or *P* < 0.001) (Fig. 3a and b), increase in the levels of cleaved caspase-3 (Fig. 3c and d), and decrease in the level of bcl-2/bax with statistical significance (Fig. 3c and e) (P < 0.05).

### Effects of sevoflurane on the proliferation of rat FNSCs

**Figure 4:**
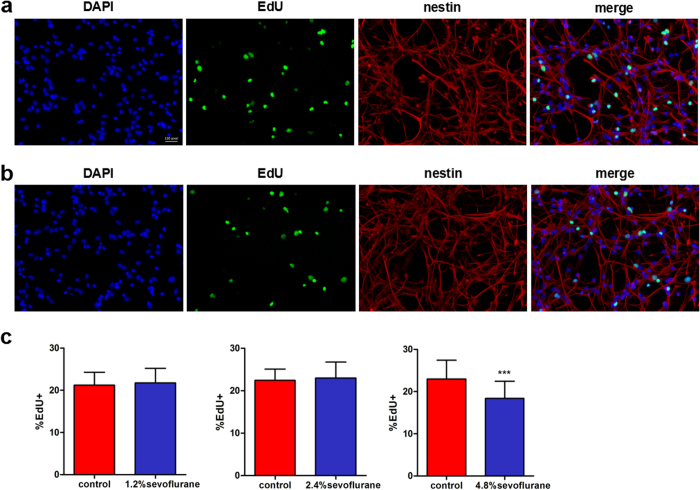
Cell proliferation was assessed immunocytochemically using the EdU incorporation assay immediately after anesthesia. (**a**) Immunofluorescence images were obtained by the EdU incorporation (*green,* Alexa Fluor 488) assay, DAPI (*blue*) nuclear staining for EdU-positive cells, and Alexa Fluor 555-stained (*pink,* Alexa Fluor 555) for nestin-marked cells and then the images were merged. (**b**) Immunofluorescence images from FNSCs exposed to 4.8% sevoflurane for 6 h. (**c**) Statistical results of EdU-positive cell ratio in FNSCs exposed to different concentrations of sevoflurane (1.2%, 2.4%, and 4.8%) for 6 h. Data were obtained from at least three separate cultures, ***P < 0.001 versus control group.

Cell proliferation was assessed immunocytochemically using EdU incorporation immediately after anesthesia. FNSCs were exposed to different concentrations of sevoflurane (1.2%, 2.4%, and 4.8%) for 6 h. Compared with the control group, the 4.8% sevoflurane group showed decreased cell proliferation indicated by EdU incorporation (***P < 0.001) (Fig. 4c), whereas the 1.2% and 2.4% sevoflurane groups showed no difference (*P* > 0.05).

### Effects of sevoflurane on the differentiation of rat FNSCs

**Figure 5:**
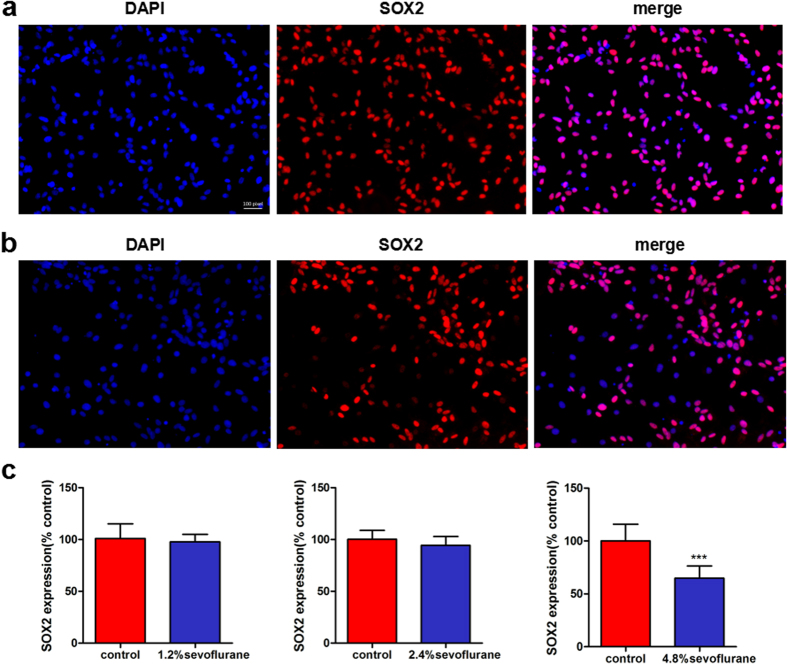
Cell differentiation was assessed immunocytochemically by SOX2 staining 24 h later. (**a**) Immunofluorescence images were obtained using SOX2 (*pink*) staining 24 h later and DAPI (*blue*) nuclear staining (undifferentiated neural stem cells), and the images were merged. (**b**) Sox2 immunofluorescence images from FNSCs exposed to 4.8% sevoflurane for 6 h. (**c**) Statistical results of SOX2-positive cell ratio in FNSCs exposed to different concentrations of sevoflurane (1.2%, 2.4%, and 4.8%) for 6 h. Data were obtained from at least three separate cultures, ***P < 0.001 versus control group.

FNSCs were exposed to different concentrations (1.2%, 2.4%, and 4.8%) of sevoflurane for 6 h. The cells were stained with SOX2 24 h later (Fig. 5a and b). The results showed that compared with the control group, the 4.8% sevoflurane group showed a decreased proportion of undifferentiated FNSCs (SOX2-positive cell ratio) on SOX2 staining (*P* < 0.0001), whereas the 1.2% and 2.4% sevoflurane groups showed no difference (*P* > 0.05) (Fig. 5c).

### Effects of sevoflurane on FNSCs via JNK pathway

**Figure 6:**
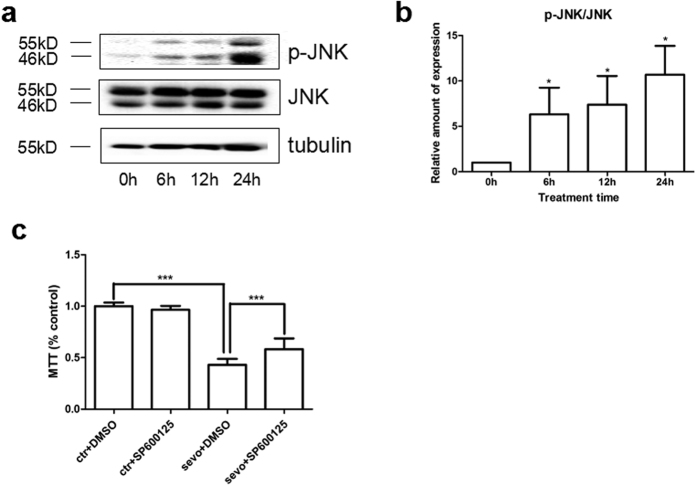
(**a**) Western blotting assay was used to detect the levels of p-JNK (46 kDa and 55 kDa) and JNK (46 kDa and 55 kDa) after different durations of exposure to 4.8% sevoflurane or control conditions. (**b**) The expression of p-JNK/JNK in FNSCs on exposure to sevoflurane for different durations. All p-JNK/JNK data are given as mean ± standard deviation from at least three separate experiments. (**c**) JNK inhibition by the specific inhibitor SP600125 rescued FNSCs from cell damage and enhanced cell viability. Data were analyzed by one-way ANOVA followed by Tukey multiple-comparison tests. *P < 0.05, ***P < 0.001.

The Western blotting assay was used to detect p-JNK (46 kDa and 55 kDa) and JNK (46 kDa and 55 kDa) levels after exposure of FNSCs to different durations of 4.8% sevoflurane (Fig. 6a). Compared with the control group, the 4.8% sevoflurane group showed a significant increase in the p-JNK/JNK ratio (*P* < 0.05) (Fig. 6b). JNK inhibition by the specific inhibitor SP600125 rescued FNSCs from a decrease in survival and upregulated cell viability (*P* < 0.001) (Fig. 6c).

## Discussion

Most clinically used general anesthetics enhance gamma-aminobutyric acid type A (GABA_A_) receptors, block *N*-methyl-D-aspartate (NMDA) receptors, or both. Sevoflurane [2,2,2-trifluoro-1-(trifluoromethyl)ethyl fluoromethyl ether] is one of the most frequently used volatile anesthetics for induction and maintenance of general anesthesia during surgery. It is particularly useful for infants and children, as it allows rapid induction and recovery and has a less irritative effect on the airway^13^. Sevoflurane has been shown to enhance GABA_A_ receptors^14^ and block NMDA receptors^15^. Although certain studies have demonstrated that sevoflurane may affect cell survival and potentiate neuronal apoptosis both *in vivo* and in *vitro*^16^,^17^, studies on the effect of sevoflurane on FNSCs have not been conducted yet. Johnson *et al*. found that a neonatal exposure to isoflurane triggered a significant neuroapoptotic response in the mouse brain^18^. Activation of the c-Jun N-terminal kinase (JNK) signaling pathway is a critical step for neuronal death occurring under several neurological conditions^19^. Previous studies have suggested that the cytotoxicity caused by sodium formate induces the activation of the JNK signaling pathway, causing caspase-dependent apoptosis. Increased levels of autophagy were observed during the process of 661 W cell damage caused by sodium formate^20^. AMN082 protects an immature brain against sevoflurane-related neurotoxicity, probably involving the extracellular signal–regulated kinases 1 and 2 (ERK1/2) and mitogen-activated protein (MAP) kinase signaling pathways^21^. A few studies investigated whether clinical doses of sevoflurane affected the survival and proliferation of cultured FNSCs via JNK pathway, which is known to play a key role in modulating bioactivities such as intracellular responses^22^.

The main findings of the present study were that subclinical doses (1.2%) of sevoflurane did not affect the proliferation and death of FNSCs, whereas exposure to higher doses suppressed proliferation and increased apoptotic cell death. Such effects occurred at clinically relevant sevoflurane doses (2.4% and 4.8%) and durations (6 h, 12 h, or 24 h). These results suggested that the survival of FNSCs depended on both the concentration and durations of sevoflurane exposure.

Moreover, many studies focused on sevoflurane-induced NSC degeneration, Yi *et al*.^23^ showed that sevoflurane inhibited embryonic stem cell self-renewal and subsequent differentiation by regulating the let-7a-Lin28 signaling pathway. Previous studies showed that sevoflurane could inhibit the central nervous system by activating the GABA_A_ receptor, resulting in cell apoptosis and degeneration of NSCs^24^. Sevoflurane may reduce neurogenesis through the Wnt–catenin signaling pathway^25^. The phosphorylation level of ERK1/2 increased after 1-h exposure to 1 or 1.5 MAC of sevoflurane in the proliferation phase, but not in the differentiation phase^26^. Sevoflurane-induced p38 activation was not affected by caspase activation. Furthermore, sevoflurane-induced apoptosis is not dependent on p38 MAP kinase activation^27^.

Prolonged treatment with high-dose sevoflurane decreased the self-renewal capacity of FNSCs by inducing apoptosis and inhibiting proliferation. Anesthetic dose and exposure duration are two important factors determining cytotoxicity. Zou *et al*.^28^ reported that exposure to ketamine (an intravenous anesthetic) for 3 h was not sufficient to induce cell death in the developing monkey brain, while a 9-h exposure at the same dose induced significant cell death^29^. Time-dependent neuronal death during anesthesia was also observed in response to isoflurane, with a minimum 2-h exposure required to induce measurable death^30^. Thus, the cytotoxic threshold concentration is dependent on the duration of exposure to several anesthetics, including sevoflurane. Excessive or prolonged gamma-aminobutyric acid type (GABA)ergic stimulation by anesthetics may induce apoptosis. Indeed, the GABA agonist ethanol activated caspase-3 in progenitor cells^31^. Alternatively, sevoflurane may suppress some neuroprotective or antiapoptotic factors such as microRNA-21^32^. One of the defining characteristics of FNSCs is proliferation. Sevoflurane is a novel volatile anesthetic, which shows less neurotoxicity than isoflurane^33^.

The effect of sevoflurane on the proliferation of FNSCs was concentration dependent; low dose did not affect proliferation, while exposure to a higher dose at the same time suppressed cell proliferation compared with the control group, which was consistent with previous studies on isoflurane^11^,^34^.

More EdU-positive cells were found under control conditions, and the plates treated with 4.8% sevoflurane for 6 h had fewer EdU-positive cells compared with time-matched control plates. Sevoflurane had no effect on the proliferation of FNSCs at low concentrations (1.2% and 2.4%), but sustained effects at higher concentrations (4.8%). The numbers of EdU-positive and SOX2-positive cells (markers for neural stem/progenitor cells) significantly decreased in FNSCs treated with 4.8% sevoflurane.

The results indicated that a higher dose of sevoflurane (4.8%) decreased the proportion of undifferentiated FNSCs, whereas sevoflurane at a lower concentration (1.2% or 2.4%) caused no difference in the number of undifferentiated cells. The Western blotting assay was used in this study to detect the levels of p-JNK and JNK. Sevoflurane exposure could increase the expression of p-JNK significantly. It was inferred that the JNK pathway played an important role in FNSC apoptosis induced by exposure to sevoflurane for different durations (6 h, 12 h, and 24 h). It proved that JNK inhibition by the specific inhibitor SP600125 rescued FNSCs from cell damage and increased neuronal survival.

Accumulating evidence indicates that signaling by the endoplasmic reticulum (ER) transmembrane protein inositol-requiring transmembrane kinase/endonuclease-1α(IRE1α) is critical for this transition from “physiological” to “apoptotic” unfolded protein response. IRE1α activation is regulated by both intra-ER and cytosolic cues. IRE1α-interacting protein ubiquitin D interacts with IRE1α in human and rodent beta cells, modulating IRE1α-dependent activation of JNK and cytokine-induced apoptosis. Long-term exposure to sevoflurane could activate JNK and induce apoptosis, especially in neural progenitor cells, resulting in the inhibition of neurogenesis *in vivo*. The present findings might raise the possibility that JNK inhibition has therapeutic potential in protecting FNSCs from the adverse effects of inhaled anesthetic.

Additional animal studies are needed to examine the effects of sevoflurane on FNSCs *in vivo* during different developmental stages. Also, patients receiving different doses of anesthetic for different durations should be examined for functional deficits. In conclusion, this study reported differential effects of different doses and durations of exposure to sevoflurane on the survival and proliferation of FNSCs. These results provided a reference for the safe use of sevoflurane anesthesia in infants and children.

Long-term exposure to sevoflurane activates JNK and induces cell damage, especially in neural progenitor cells, inhibiting neurogenesis. The present findings raise the possibility that JNK inhibition has therapeutic potential in protecting FNSCs from the adverse effects of drugs. Overall the present findings unveil a novel JNK pathway and function, which is likely to play a role in inhaled anesthetic-induced apoptosis. The research provided evidence for the JNK blocker as a potent neurotoxicity therapeutic candidate acting via JNK-dependent pathways.

## Additional Information

**How to cite this article**: Yang, Z. *et al*. Sevoflurane decreases self-renewal capacity and causes c-Jun N-terminal kinase–mediated damage of rat fetal neural stem cells. *Sci. Rep.* **7**, 46304; doi: 10.1038/srep46304 (2017).

**Publisher's note:** Springer Nature remains neutral with regard to jurisdictional claims in published maps and institutional affiliations.
